# A Meta-Analysis on Clinical Outcomes of Ceftolozane versus Piperacillin in Combination with Tazobactam in Patients with Complicated Urinary Tract Infections

**DOI:** 10.1155/2022/1639114

**Published:** 2022-08-08

**Authors:** Muhammad Waqas Saeed, Syed Wasif Gillani, Rana Kamran Mahmood, Prasanna Vippadapu, Mian Waqar Hussain, Hassaan Anwer Rathore

**Affiliations:** ^1^Department of Pharmacy Practice, College of Pharmacy, Gulf Medical University, Ajman, UAE; ^2^Department of Pharmacy, Rashid Hospital, DHA, Dubai, UAE; ^3^Department of Pharmacy, Response Plus Medical, Abu Dhabi, UAE; ^4^Department of Pharmaceutical Sciences, College of Pharmacy, QU Health, Qatar University, Doha, Qatar

## Abstract

**Objective:**

To evaluate efficacy and adverse events of ceftolozane/tazobactam in complicated UTI including acute pyelonephritis.

**Method:**

Databases that include PubMed, Embase, Scopus, and TRIP were searched. All randomized controlled trials and cohort studies were considered for the study. Statistical analysis was done using a fixed effects model, and results were expressed in proportion for dichotomous data and risk ratio for continuous data with 95% confidence intervals (CI).

**Results:**

A clinical cure of ceftolozane/tazobactam was found to be 92% with 95% CI of 90-94 while that of piperacillin/tazobactam was only 78% (95% CI, 74-82) in patients with complicated UTI. Microbiological eradication was still higher in the ceftolozane/tazobactam group (83%, 95% CI 81-88) when compared with piperacillin/tazobactam (63% 95% CI, 58.77-65.2). Ceftolozane/tazobactam was more effective in the treatment of complicated urinary tract infections other than acute pyelonephritis as compared to piperacillin/tazobactam (RR = 1.21, 95% CI, 1.07-1.23). Serious adverse events were found comparable in both groups (RR = 1.15, 95% CI, 0.64-2.09).

**Conclusion:**

The analysis showed that ceftolozane/tazobactam has better clinical outcomes including cure rates and low resistance for the treatment of complicated urinary tract infection.

## 1. Introduction

Infection of the urinary tract is among the most common types of infections that affect a large number of populations [1]. Urinary tract infection (UTI) comprises urethritis, cystitis, prostatitis, and pyelonephritis. Furthermore, UTI can be divided as complicated and uncomplicated infections. It is important to differentiate between uncomplicated and complicated infections. Uncomplicated infections are more common in females in their childbearing age than in males, whereas complicated infection (cUTI) is equally common in both males and females [2]. Complicated UTI is occurring only in patients who have structural, functional, or surgical abnormalities such as renal calculi, renal transplant, or catheter insertion [3].

The most common route for the microorganism to reach the upper urinary system is by the urethra although hematogenous lymphatic spread is also there [4]. Bacteria that cause complicated infection include *E. coli* (but accounting less than 50% infections), Proteus spp., K. pneumoniae, Enterobacter spp., P. aeruginosa, staphylococci, and enterococci [5]. Enterococcus species especially vancomycin resistant E. faecalis and E. faecium are becoming more common in hospitalized patients [6]. Candida species are also common in hospitalized patients having complicated UTI [7].

Variation in complication factors and susceptibility pattern of microorganisms in complicated UTI patients makes it difficult for the selection of antimicrobial therapy. Thus, empiric treatment with an unproven regimen is used for such infections [8]; selection of antimicrobials in patient with critical illness is of utmost important as we need the best possible result with least risks [9]. Selection of antimicrobials depends on the type of microorganisms causing complicated UTI. In critically ill patients, intravenous fluoroquinolones, aminoglycosides with or without ampicillin, and extended spectrum cephalosporin with or without aminoglycosides are used as empiric therapy [10, 11]. Other antibiotics that are used for complicated UTI are aztreonam and beta-lactam inhibitor combinations (such as piperacillin/tazobactam, ceftazidime/avibactam, and ceftolozane/tazobactam) [12, 13]. Carbapenems (imipenem, doripenem, meropenem, or ertapenem) are also used in the treatment of complicated UTI [11, 14, 15]. In case resistant gram-positive uropathies are expected, vancomycin, distamycin, or linezolid should be added to the treatment [16–18]. In several studies, it was found that ceftolozane/tazobactam has potent activity against gram-negative bacteria causing urinary tract infection [19–21].

The increase in prevalence of bacteria resistant to the current antibiotic therapy and limited new antibiotics is very well documented [22]. Selection of antimicrobials is sometime inappropriate in patients with pathogenic infection with high risk of developing resistance [23]. Thus, it is highly important to select the best suitable antibiotic for the treatment of infection. High prescription load of piperacillin/tazobactam has increased the risk of inappropriate therapy as well as developing resistance [24].

Availability of different beta-lactams underlines the need of finding the best beta-lactam for the treatment for complicated urinary tract infection. The purpose of the study is to assess the comparison in efficacy and safety of ceftolozane/tazobactam and piperacillin/tazobactam for the treatment of complicated urinary tract infection.

High consumption of piperacillin/tazobactam has increased the chance of inappropriate use leading to high risk of developing the resistance. Thus, it is necessary to find the best possible therapy to eradicate pathogens completely to avoid developing resistance. The purpose of the study is to compare ceftolozane/tazobactam against piperacillin/tazobactam for the treatment of complicated urinary tract infection including acute pyelonephritis for resolution of clinical symptoms with least development of resistance and low risk of adverse events. The aim of this review was to assess clinical outcomes of ceftolozane/tazobactam and piperacillin/tazobactam when used for the treatment of complicated urinary tract infections. Such outcomes include clinical outcomes, bacterial eradication, resistance, and adverse events, thus selecting the better antibiotic for the treatment of complicated UTI.

## 2. Materials and Methods

### 2.1. Criteria for Quantitative Evidence Development

#### 2.1.1. Types of Studies

Randomized control trials and longitudinal cohort studies recorded the clinical outcomes of piperacillin/tazobactam and ceftolozane/tazobactam that were used in the treatment of complicated urinary tract infections. Where studies included patients with other infections (e.g., pneumonia and sepsis), studies were included if results for therapy of UTI could be extracted separately.

#### 2.1.2. Types of Participants

To this meta-analysis, complicated UTI was defined as symptomatic UTI having clinical symptoms of fever, pyuria, flank pain, and costovertebral angle tenderness. Such symptoms are associated with infections that extended beyond the bladder such as pyelonephritis. Studies that included patients with mild infection like cystitis were excluded. Complicated UTI include patients with preexisting kidney disease such as obstruction, neurogenic bladder, azotemia due to renal disease, urinary retention due to benign prostate hypertrophy, or chronic catheterization.

Studies including complicated and uncomplicated UTI were excluded under the assumption that underlying bacterial species are different in both complicated and uncomplicated infections. Studies including only uncomplicated UTI were also excluded under the same assumption. Studies having patients with nosocomial infections were also included in the review. There were no age or sex restrictions in the selection of participants.

Types of interventions are as follows:

1. Administration of piperacillin/tazobactam
2. Administration of ceftolozane/tazobactam

Types of outcome measures are as follows:

1. Cure rates (defined as no clinical signs; bacteriological cure rate defined as eradication of bacteria and combined clinical and bacteriological cure rate defined as no clinical signs and eradication of bacteria): (a) under therapy, (b) at the end of therapy, and (c) after an interval
2. Clinical cure cUTI and acute pyelonephritis
3. Antimicrobial resistance (defined as (a) for ceftolozane/tazobactam MIC > 32 mg/dL and (b) for piperacillin/tazobactam MIC = 16 mg/dL)
4. Adverse events

### 2.2. Search Methods for Identification of Studies

The meta-analysis was synthesized with the Preferred Reporting Items for Systematic Reviews and Meta-analysis (PRISMA) guidelines [25]. Four international electronic databases (PubMed, Cochrane, Springer, and Google Scholar) were comprehensively searched from initiation up to December 4^th^, 2020, for all studies assessing the clinical outcomes of piperacillin/tazobactam and ceftolozane/tazobactam in the treatment of complicated urinary tract infection.

### 2.3. Keywords and Searching Details

The search was performed by combination of the following search terms using the Boolean operators “OR and/or AND”: “complicated urinary tract infection”, “pyelonephritis”, “nosocomial urinary tract infection”, “Piperacillin”, “Tazobactam”, “Piperacillin/tazobactam”, “ceftolozane”, and “ceftolozane/tazobactam” (Supplementary Fig 1). We restricted the literature search to English language reports and human subjects. Additional trials were identified by reviewing the reference lists of eligible studies and review articles.

PubMed: PubMed search was done using the combinations of keywords mentioned above. Filters of randomized controlled trials and English language were selected to limit the search. Filter for publication year was not used.

Springer: search was done using the advanced search option in Springer using Boolean operators. Filters of clinical trials, internal medicine, and English language were selected to reduce the search trial list.

Cochrane: search was done with above terms, and filters of randomized controlled trials, infectious disease, and urinary tract infections were applied.

Google Scholar: search was done with above mentioned terms using Boolean operators. No filters were applied during search.

### 2.4. Data Collection

The search strategy as described above was done by two authors with the help of a supervisor. Titles and abstract were screened, and where necessary, the full text was assessed. Studies reported in language other than English were excluded. Studies with unclear presentation or incomplete data were also not included in meta-analysis.

### 2.5. Data Extraction Process

Data was extracted using standard data extraction sheets. Quality of studies was assessed using the Cochrane collaboration tool ROB2 without blinding to authorship [26]. The items assessed were allocation sequence, allocation concealment, blinding and availability of outcomes of participants, outcome measurement, multiple eligible outcome, and use of intention-to-treat analysis. The allocation concealment was considered adequate if the randomization method would not allow the investigator or participants to know or influence to which intervention group the patient were involved before the beginning of the study. Blinding was divided into participants, investigator, and assessor. Measurement outcomes mean that outcomes were recorded for all participants or not. Multiple eligible outcomes represent that either outcomes were recorded using time options, scales, or definitions within the outcome domain or not. If multiple outcomes are noted, the risk of bias increases. To classify a study as intention-to-treat analysis, study assessment had to confirm that all randomized patients were analyzed according to the randomization schedule, while longitudinal cohort trials were assessed for quality using the NIH quality assessment tool for before-after (pre-post) studies with no control group [27].

### 2.6. Statistical Analysis

For statistical analysis, dichotomous outcomes were expressed as proportion with 95% confidence interval (CI). For continuous outcomes, the proportion was used, also with 95% CI. In both cases, data were pooled using the fixed effects model. Heterogeneity was assessed using a chi-square statistic with an alpha of 0.1 for statistical significance and the *I*^2^ statistic. *I*^2^ values of 25%, 50%, and 75% correspond to low, medium, and high levels of heterogeneity. There were insufficient studies to examine publication bias.

## 3. Results

### 3.1. Description of Studies

**Figure 1:**
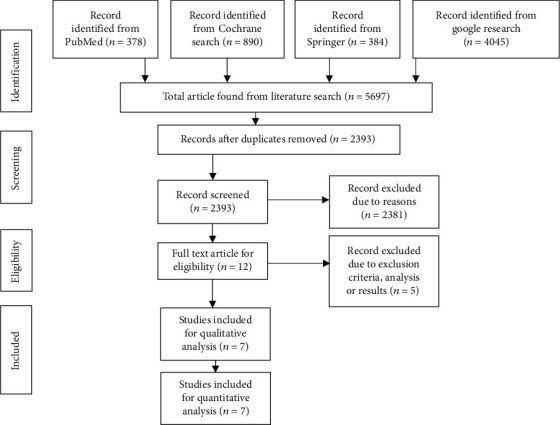
PRISMA flow chart.

A systemic literature search was done using the search strategy developed above, and 5679 studies were found using the selected keywords. A total of 3286 were duplicate articles, while 2381 articles were excluded as they were not related to the study at all. The remaining 19 articles were assessed for eligibility out of which 8 articles were excluded as they were either editorial analysis or meta-analysis (5), lack comparison (2), or have a duplicate database (2) (Figure 1) [28–34].

Studies were conducted in 30 countries: South Korea, Belarus, Brazil, Greece, Hungary, Peru, Poland, Czech Republic, Italy, Romania, Slovakia, Romania, United States, Chile, Columbia, Croatia, Estonia, Georgia, Germany, India, Israel, Latvia, Mexico, Moldova, Russia, Serbia, Slovenia, South Africa, Spain, and Thailand. Studies were published in the English language between 2012 and 2019 (Table 1).

A total of nine hundred and seventy-seven participants were recruited from seven studies. Among these, 2 studies included patients above sixty-five years making as total of 203 participants, while five studies showed the mean age of participants.

All studies required clinical signs and symptoms of complicated UTI and pyelonephritis (fever > 38, chills, rigor, nausea or vomiting, dysuria, lower abdominal pain, and pyuria) and positive urinalysis. In all studies, a positive urine culture before treatment was a prerequisite. Seo et al. recruited participants who were having healthcare-associated UTI. Five studies used fever and pain as sign of severity and laboratory analysis (elevated WBC) or specified that the clinical condition had to require parenteral antibiotics. In one study, clinical signs and symptoms were not defined. Patients with negative urinalysis were excluded. Studies that only reported pooled data instead of reporting single study and explicitly included patients with complicated UTI were excluded from this review.

#### 3.1.1. Interventions

The keys drugs included in the first study were ceftolozane/tazobactam versus levofloxacin, which were analyzed. Ceftolozane/tazobactam 1.5 g was administered intravenously every eight hours, whereas levofloxacin 750 mg was intravenously administered once daily. Duration of treatment in two treatment groups was between 7 and 14 days [29].

Analysis of comparison of meropenem/tazobactam against piperacillin/tazobactam therapy was performed in the second study. 4 g meropenem/vaborbactam in 250 mL of normal saline was infused over three hours every eight hours. Piperacillin/tazobactam 4.5 g was administered intravenously in 100 mL normal saline every eight hours. The therapy was switched to levofloxacin 500 mg oral tablet after the administration of 15 doses in each group, if clinically indicated. The duration treatment in each group was 15 days [30].

Comparison of fosfomycin and piperacillin/tazobactam was studied in the third study. 6 g of fosfomycin is fused intravenously every eight hours in group one while piperacillin/tazobactam 4.5 g every eight hours in group 2. The duration of treatment in each group was 7 to 14 days [31].

Study four analyzed piperacillin/tazobactam, cefepime, and ertapenem therapy for complicated UTI. Piperacillin/tazobactam 4.5 g IV given every 6 hours, ertapenem 1 g IV once daily, and cefepime 2 g IV every 12 hours were given. The duration of each treatment group was 10 to 14 days [33].

The key drug analyzed in two longitudinal cohort studies was ceftolozane/tazobactam. The dose was the same in both studies that 1.5 g of ceftolozane/tazobactam was given intravenously every eight hours.

#### 3.1.2. Outcomes

Outcomes varied in different studies, so it was not always feasible to combine results. Even the primary outcome was defined differently. Cure rates were presented as clinical or bacteriological or combined clinical and bacteriological cure rates; the outcome was assessed during therapy or at the end of therapy. Three studies presented adverse events, while one study did not provide any information regarding adverse events (Table 2).

**Figure 2:**
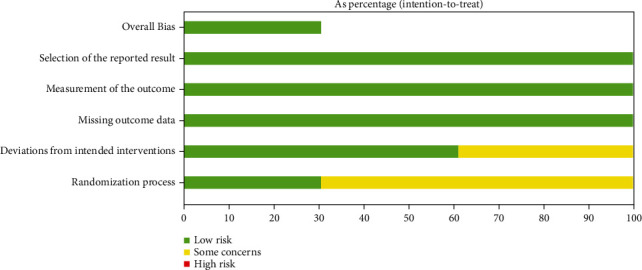
Quality assessment of screened randomized controlled trials.

Quality assessment was done using ROB2 of screened articles. Overall, the risk of bias for the articles was low. But there were some concerns in the randomization process in two studies while in one study, deviation from intended interventions was also noted (Figure 2). Quality assessment of the longitudinal cohort study was done using the NIH quality assessment tool for before-after (pre-post) studies with no control group [27].

#### 3.1.3. Effect of Intervention

**Figure 3:**
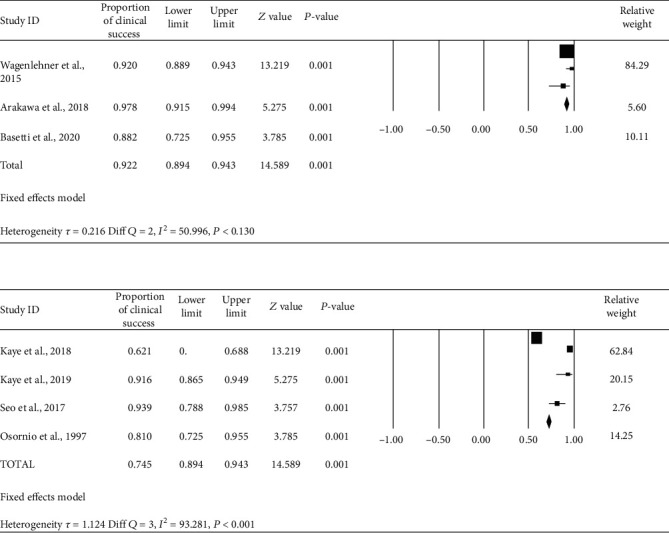
Forest plot of clinical cure of ceftolozane/tazobactam and piperacillin/tazobactam in patients with cUTI. Black squares indicate proportion, and horizontal lines indicate 95% CI.

*(1) Clinical Cure Rates of Ceftolozane/Tazobactam and Piperacillin/Tazobactam*. The review indicates that the clinical treatment success rate at the end of treatment with ceftolozane/tazobactam from three studies was 92% (95% CI, 90-94%) in the treatment of complicated UTI, whereas the clinical treatment success rate of piperacillin/tazobactam was 78% (95% CI, 74-82%) (Figure 3). The risk ratio of 1.18 (95% CI, 1.12-1.25) was found to indicate that therapy failure is more in the piperacillin/tazobactam group. We did within-group analysis to evaluate reason heterogeneity, and Figure S1 represents the funnel plot by removing studies with publication bias-reduced heterogeneity.

**Figure 4:**
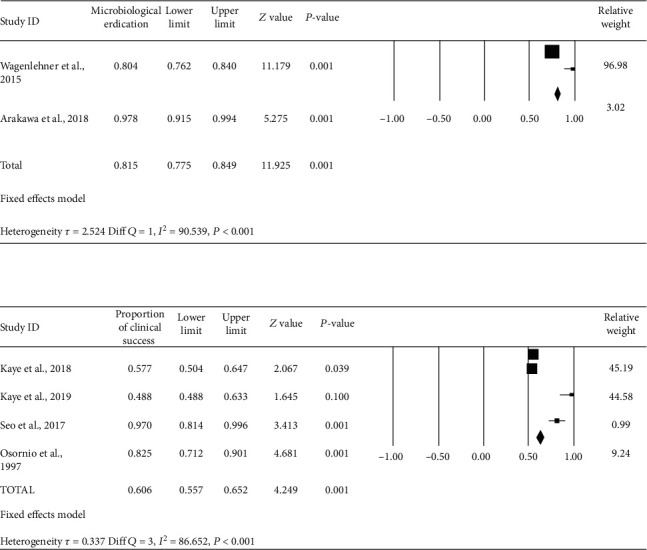
Forest plot of microbiological eradication of ceftolozane/tazobactam and piperacillin/tazobactam in patients with cUTI. Black squares indicate proportion, and horizontal lines indicate 95% CI.

*(2) Microbiological Eradication of Pathogens by Ceftolozane/Tazobactam and Piperacillin/Tazobactam*. Studies showed proportion of microbiological eradication of pathogens by ceftolozane/tazobactam, indicating the pooled microbiological eradication of 85% with 95% CI (81-88%). In pooled microbiological eradication rates of piperacillin/tazobactam, percentage success was only 63% (95% CI 58.77-67%) (Figure 4). The risk ratio of 1.35 with 95% CI (1.24-1.46) again shows that the risk of failure is more in the piperacillin/tazobactam group. Within-group analysis was done for the evaluation of reasons for heterogeneity. Figure S2 shows the funnel plot after removal of studies with publication bias thereby reducing heterogeneity.

**Figure 5:**
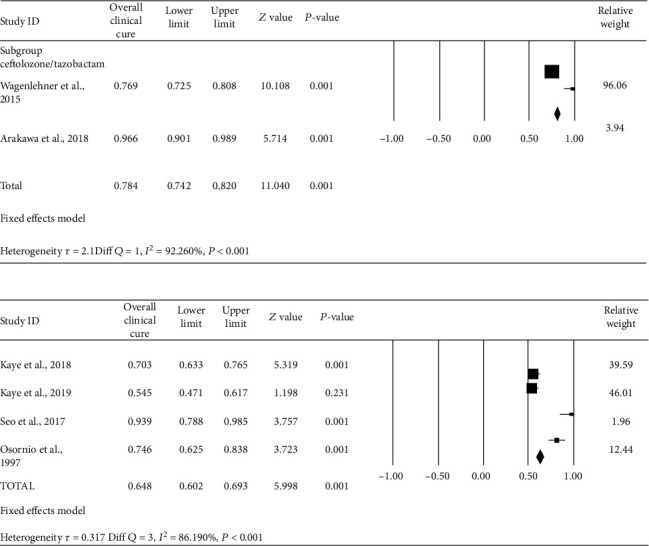
Forest plot for overall clinical success after 28 days of ceftolozane/tazobactam and piperacillin/tazobactam in patients with cUTI. Black squares indicate proportion, and horizontal lines indicate 95% CI.

*(3) Overall Clinical Success Rate at the End of 28 Days of Treatment*. Finding indicates the overall clinical success rate in the treatment of complicated UTI by ceftolozane/tazobactam and piperacillin/tazobactam. Pooled data of overall clinical success rates showed 82% of success rate with 95% CI (78-85%), while the overall clinical success rate for piperacillin/tazobactam was 66% (95% CI 61-70%) (Table 3). Analysis is done within group for the evaluation of heterogeneity.  Figure 5(s) represents the funnel plot after removal of studies with publication bias showing reduction in heterogeneity.

*(4) Clinical Cure Rate in cUTI*. The clinical cure rate in patients with complicated UTI excluding acute pyelonephritis was found to be 73% in ceftolozane/tazobactam where in 82 patients out of 118, the cure was successful, as compared to the piperacillin/tazobactam group where the success rate of clinical cure was 57%, consisting of 70 out of 122 infected patients (Table 2).

*(5) Clinical Cure Rate in Acute Pyelonephritis*. The number of patients with acute pyelonephritis that had successful clinical cure was 273 out of 350, showing a clinical cure successful rate of 78%, whereas patients in the piperacillin/tazobactam group showed a success rate of 80% with 157 successfully treated patients out of 194 (Table 3).

*(6) Microbiological Eradication of E. coli*. The percentage of Enterobacteriaceae was 88% in the ceftolozane/tazobactam group which was higher when compared with that in the piperacillin/tazobactam group where the percentage of *E. coli* eradication was noted to be 78% (Table 2).

*(7) Resistance to Antibiotics*. The rate of resistance was found to be quite low in the ceftolozane/tazobactam group of 2.7% as compared to the piperacillin/tazobactam group where the rate of resistance was 10% (Table 3).

*(8) Adverse Events*. All adverse events recorded were found to be higher in the ceftolozane/tazobactam group (almost 38%), whereas in the piperacillin/tazobactam group, the recorded events were 33%. Serious drug events associated with study drugs were 4% in the ceftolozane/tazobactam group while in the piperacillin/tazobactam group, serious drug events were 3.7%.

## 4. Discussion

The review showed the clinical cure rate of both beta-lactam/beta-lactamase inhibitors with 17% higher clinical cure rates with the ceftolozane/tazobactam group where overall treatment success was 92% when compared with the piperacillin/tazobactam group with a clinical cure rate of 78%. The risk ratio also signifies that there is a chance of therapy success with ceftolozane/tazobactam when compared with piperacillin/tazobactam. This result is also supported by another study that reported high clinical cure rates in cUTI [35]. Another study showed similar results where ceftolozane/tazobactam was more effective in the treatment of infection caused by Pseudomonas aeruginosa than piperacillin/tazobactam [36].

Microbiological eradication was also higher in the ceftolozane/tazobactam group where the rate of microbiological eradication was noted to be 85% almost 22% higher than the control group where the success rate was only 63%. Thus, at the end of treatment, patients with no pathogens were more in the ceftolozane/tazobactam group. The results are also confirmed by a study which showed higher with ceftolozane/tazobactam even in patients with carbapenem-resistant infection which recorded their experience [37]. Another study supports the result of ceftolozane/tazobactam superiority in microbiological eradication of *E. coli* and *P. aeruginosa* [38]. Furthermore, the microbiological eradication of ceftolozane/tazobactam was found to be very high in one meta-analysis (OR 1.31, 95% CI, 0.42-4.10; *I*^2^ = 37%) [39].

The study findings showed that overall clinical cure rates after 28 days were also found to be higher in the comparative group where success was 82%, higher by 16% than that in the control group. In the ceftolozane/tazobactam group, the risk of relapse was lower than that in the piperacillin/tazobactam group after 28 days of treatment therapy. A study has reported sixty percent mortality rates after twenty-eight days in patients with UTI in the group of piperacillin/tazobactam [40]. Pooled analysis of the ASPECT trial also confirmed better overall cure rates with ceftolozane/tazobactam [41].

The treatment of complicated UTI other than acute pyelonephritis was found to be better with ceftolozane/tazobactam as compared to piperacillin/tazobactam where 77% of total patients in experimental groups received successful treatment with ceftolozane/tazobactam (RR 1.21), while treatment of acute pyelonephritis was found to be similar in both the experimental (78%) and control groups (80%), with RR of 0.97(95% CI, 0.89-1.05).

In ceftolozane/tazobactam, the rate of resistance was much lower of almost 2.7%, that is, four times lower than that in the piperacillin/tazobactam group. Thus, there is a high of development of resistance against piperacillin/tazobactam as compared to ceftolozane/tazobactam. A study recorded the efficacy and resistance of ceftolozane/tazobactam against the comparators including piperacillin/tazobactam that were found to be resistant. Organisms like *K*. *pneumonia* (100%), *Enterobacter* (38.9%), *P aeruginosa* (37.4%), and *E. coli* (17.9%) developed resistance against piperacillin/tazobactam. It was found that ceftolozane/tazobactam was effective even for such resistant strains [42, 43].

General adverse events were noted more in the experimental group than in the control group. A risk ratio of 5.11 with 95% CI 3.01-8.68 clearly indicates that there are more adverse events in patients who received ceftolozane/tazobactam as compared to those receiving piperacillin/tazobactam. Serious adverse events were also higher in the experimental group than in the control (RR 1.15, 95% CI 0.64-2.09). A systematic review reported that the most common adverse event with ceftolozane/tazobactam was hypokalemia that was 4.2% out of 48 evaluable cases [43, 44]. Another meta-analysis showed a similar risk ratio of 1.16 with 95% CI 0.67-1.99 serious adverse events related to ceftolozane/tazobactam [39, 44].

Limitations present in this meta-analysis should be noted. The clinical cure rates, microbiological eradication, and overall cure rates were derived from cohorts with different methods to measurements (i.e., cure rates after 4 days or cure rates after seven days). High heterogeneity was noted due to difference in effect size, study design (retrospective or prospective, open label), or patient with infections from different pathogens.

## 5. Conclusion

The meta-analysis concluded that ceftolozane/tazobactam has better clinical outcomes in patients with complicated urinary tract infections, except for acute pyelonephritis. So the use of ceftolozane/tazobactam in acute pyelonephritis should be avoided. The risk of resistance is also low in the ceftolozane/tazobactam group, therefore reducing the stay of patient in the healthcare facility. There are significantly high rates of side effects among ceftolozane/tazobactam compared to piperacillin/tazobactam; however, these side effects did not contribute to severe morbidity or mortality.

## Figures and Tables

**Table 1:** Characteristics of study.

	Wagenlehner et al., 2015	Kaye et al., 2018	Kaye et al., 2019	Seo et al., 2017	Arakawa et al., 2015	Osornio et al., 1997	Basetti et al., 2020
Methods	Prospective RCT Double blind Computer generated randomization Intention to treat	Prospective RCT Double blind Dynamic randomization algorithm and voice/web system Intention-to-treat analysis	Prospective RCT Randomization was by ratio 1 : 1 Intention-to-treat analysis	Prospective RCT Randomization was done at each center A laboratory center monitored the balance in sample size Intention-to-treat analysis	Prospective Longitudinal cohort study No comparative open-label study	Retrospective multicenter study where patients were treated with piperacillin/tazobactam	Prospective Longitudinal cohort study No comparative open-label study

Participants	Inclusion criteria Number: 398 (group 1), 402 (group 2) Gender (M/F): unknown Age: >18 years Clinical symptoms of severe UTI requiring parenteral antibiotic therapy and a positive (fever > 38, dysuria, flank pain, costovertebral tenderness, nausea, or vomiting) urine culture (pyuria: white blood cell count > 10/*μ*L in unspun urine or ≥10/*μ*L per high power field in spun urine) Exclusion criteria Cases of allergy, severe underlying disease, renal impairment, pregnancy, urinary tract obstruction, concomitant infection, receipt of any dose prior to trial, intractable urinary tract infection, confirmed fungal urinary tract infection, suspected or confirmed prostatitis or intrarenal abscesses, permanent indwelling bladder catheter, immunocompromised conditions	Inclusion criteria Number: 233 (group 1), 231 (group 2) Gender (M/F): unknown Age: >18 years Clinical symptoms of severe UTI requiring parenteral antibiotic therapy and a positive (fever > 38, dysuria, flank pain, costovertebral tenderness, nausea, or vomiting) urine culture (pyuria: white blood cell count > 10/*μ*L in unspun urine or ≥10/*μ*L per high power field in spun urine) Exclusion criteria Cases of allergy, severe underlying disease, renal impairment, pregnancy, urinary tract obstruction	Inclusion criteria Number: 178 (group 1), 169 (group 2) Gender (M/F): unknown Age: >18 years Clinical symptoms of severe UTI requiring parenteral antibiotic therapy and a positive (fever > 38, dysuria, flank pain, costovertebral tenderness, nausea, or vomiting) urine culture (pyuria: white blood cell count > 10/*μ*L in unspun urine or ≥10/*μ*L per high power field in spun urine) Exclusion criteria Cases of allergy, severe underlying disease, renal impairment, pregnancy, urinary tract obstruction, nonrenal source of infection, severe sepsis, immunosuppressive medications, uncomplicated UTI	Inclusion criteria Number: 33 (group 1), 33 (group 2), 6 (group 3) Gender (M/F): 30 F (group 1), 26 F (group 2), 3 F (group 3) Age: >18 years Hospital-associated UTI Clinical symptoms of severe UTI requiring parenteral antibiotic therapy and a positive (fever > 38, dysuria, flank pain, costovertebral tenderness, nausea, or vomiting) urine culture (pyuria: white blood cell count > 10/*μ*L in unspun urine or ≥10/*μ*L per high power field in spun urine) Exclusion criteria Presence of suspicious or confirmatory infectious foci other than urinary tract infection, any use of antibiotics within seven days prior to recruitment for any reasons, any complicating urinary factors which could not be effectively treated during trial (obstruction, suspected or confirmed prostatitis, epididymitis), indwelling urinary catheters expected to remain in place after therapy has been completed, and need for renal replacement therapy	Inclusion criteria Number: 115 Males or gender (M/F) cUTI or acute pyelonephritis who need hospitalization and need antibacterial IV therapy Exclusion criteria History of recent or recurrent gram-positive organism UTI suggesting colonization, moderate or severe hypersensitivity, or allergic reaction to any beta-lactam antibacterial, receiving probenecid, has received any amount of potentially therapeutic antibacterial therapy after collection of the pretreatment baseline urine culture, complete, permanent obstruction of the urinary tract, confirmed fungal urinary tract infection at time of randomization	Inclusion criteria Number: 79 Gender (M/F) Data were recorded: age and sex, underlying diseases according to Charlson comorbidity index, type of infection, presence of sepsis or septic shock at the time of the infection, susceptibility pattern of ESBL-E isolates, date of start and end of C/T therapy, source control of infection, when applicable, other antibiotics administered before, concomitant to, and after C/T therapy, reasons for C/T use, dosage(s) of C/T and length of therapy, adverse events (AEs), clinical outcome, and recurrence of infection	Inclusion criteria Number: 153 Gender (M/F) Data were recorded: age and sex, with clinical and microbiological evidence of UTI caused by microorganism susceptible to piperacillin/tazobactam, have clinical symptoms (fever > 38, chills, flank pain, dysuria costovertebral tenderness, nausea, or vomiting) Urine culture (pyuria: >5WBC/HPF, peripheral white blood count ≥ 10,000 mm^3^ with >5% bands) Exclusion criteria Patient allergic to beta-lactams or beta-lactamase inhibitors, an FiO_2_ 60% at maintaining aerial hemoglobin oxygen saturation to 90%, septic shock, endoscopic prostatic resection, use of antibiotic within 72 hours, presence of resistance strain, thrombocytopenia, creatinine clearance less than <40 mL/min, peritoneal dialysis, serum concentration of aminotransferase or bilirubin to be twice the normal value

Interventions	Treatment group 1 Ceftolozane/tazobactam 1.5 g every 8 hours IV for 7 days Treatment group 2 Levofloxacin 750 mg once daily IV for 7 days	Treatment group 1 Meropenem vaborbactam 4 g in 250 mL normal saline IV administer over 3 hours every 8 hours followed by 100 mL saline over 30 minutes every 8 hours Treatment group 2 Piperacillin-tazobactam 4.5 g in 250 mL normal saline IV administer over 30 minutes every 8 hours Levofloxacin 500 mg once daily for 10 days after 15 doses of piperacillin-tazobactam, if clinically indicated Cointervention Both groups received levofloxacin 500 mg oral once daily after 15 doses of each intervention	Treatment group 1 Fosfomycin 6 g IV administered every 8 hours for 7 to 14 days Treatment group 2 Piperacillin-tazobactam 4.5 g IV administered every 8 hours for 7 to 14 days	Treatment group 1 Piperacillin/tazobactam 4.5 g q 6 hours for 10-14 days Treatment group 2 Ertapenem 1 g, IV q 24 hours for 10-14 days Treatment group 3 Cefepime 2 g, IV q 12 hours for 10-14 days	Treatment group 1.5 g (ceftolozane 1 g/tazobactam 0.5 g) administered as an intravenous (IV) infusion every 8 hours for 7 days	Treatment group Piperacillin-tazobactam 4.5 g IV administered every 8 hours for 10 to 14 days	Treatment group Ceftolozane 1 g/tazobactam 0.5 g administered as intravenous infusion every 8 hours

Outcomes	Clinical and microbiological eradication outcomes at test of cure visit in the mMITT population (test of cure visit 7 days after the completion of study drug administration) Clinical and microbiological eradication outcomes at test of cure visit in the microbiological evaluation population (test of cure visit 7 days after the completion of study drug administration)	(1) Proportion of participants in the microbiological modified intent-to-treat (m-MITT) population who achieved overall success at the end of intravenous treatment visit (time frame: EOIVT (days 5-14)) (2) Proportion of participants in the m-MITT population who achieved a microbiologic outcome of eradication at the test of cure visit (time frame: test of cure (TOC) (days 15-23)) Proportion of participants in the microbiological evaluable (ME) population who achieved a microbiologic outcome of eradication at the TOC visit (time frame: TOC (days 15-23))	(1) Number of patients with an overall success (time frame: TOC visit (day 19)) (2) Number of patients with a response of clinical cure in various protocol populations (time frame: TOC visit (day 19)) (3) Number of patients with a response of microbiologic eradication (time frame: TOC visit (day 19))	(1) Clinical improvement rate (time frame: 3-5 days after treatment) (2) Microbiological eradication rate (time frame: 10-14 days after treatment)	(1) Percentage of participants with microbiological response (eradication, persistence, or indeterminate) at test of cure (TOC) (time frame: up to 14 days after the first dose of study drug (up to 14 days)) (2) Percentage of participants with adverse events (AEs) (time frame: from time of the first dose of study drug until the end of follow-up (up to 42 days)) (3) Percentage of participants discontinuing study drug due to AEs [time frame: Up to 7 days after the first dose of study drug (up to 7 days)]	(1) Clinical failure was defined as either lack of clinical response or recurrence or attributable mortality due to ESBL-E infection (2) Clinical failure was confirmed by (a) 30-day mortality, (b) ongoing fever after 5 days of therapy, (c) persistence of leukocytosis after 5 days of therapy, and (d) presence, after 5 days of therapy, of clinical signs of infection that could not be attributed to causes other than ESBL-E infection	(1) Patients treated with piperacillin/tazobactam are said to be cured if the patient was asymptomatic without any evidence of active infection at the end of treatment (time frame: 5-9 days after treatment for early evaluation and 4-6 weeks after termination of therapy) (2) Improvement of a posttherapy evaluation but without complete resolution of symptoms (3) Relapse, initial improvement during first 3-4 days of treatment followed by deterioration during therapy or at the posttreatment evaluation (5-9 days)

Notes	Of 1083 patients who met the inclusion criteria, 800 were enrolled (16 received no study drug) Two hundred sixty-eight patients were excluded after randomization because urine culture was negative Thirty-one (group 1) and forty-two (group 2) were lost to follow-up Three in each group were without cUTI diagnosis Seven (group 1) and five (group 2) received nonstudy antibiotic Four (group 1) and nine (group 2) did not adhere to study treatment Three (group 1) and one (group 2) catheter not removed by the end of treatment	Of 585 patients who met the inclusion criteria, 550 were enrolled (23 did not met inclusion and exclusion criteria, 7 withdrew consent, and 5 other reasons) Five patients were excluded after randomization because they did not receive study drug Thirty-one (group 1) and forty-two (group 2) were lost to follow-up Twenty-three (group 1) and thirty-eight (group 2) discontinued treatment Fourteen (group 1) and twenty-three (group 2) discontinued study Eighty (group 1) and ninety-one (group 2) no baseline pathogens	Of 465 patients who met the inclusion criteria, 464 were enrolled (1 did not get the study drug) Twelve (group 1) and two (group 2) did not complete the study Fourteen (group 1) and nine (group 2) discontinued the study	(1) If any participant receiving randomized drug dropped out, the drug was given to the next participant (2) Recruitment in group 3 was stopped after 6 participants due to high risk of therapy failure	Out of 115 patients who met the inclusion criteria, 112 completed the study (1 withdrew consent while 2 physician decided)	No dropouts were there as it was a retrospective study	Out of 79 patients, 61 patients were included in clinical and bacteriological evaluation. 6 were excluded due to abnormal baseline aminotransferase, 3 were excluded due to inappropriate drug regimen, 1 was excluded due to the presence of resistant pathogen, 2 were excluded due to the previous antibiotic use

**Table 2:** The quality assessment tool for before-after (pre-post) studies with no control group: scores of included studies.

	Scale items^a^												
	1	2	3	4	5	6	7	8	9	10	11	12	Score
Arakawa et al. (2019)	Y	Y	Y	Y	N	Y	Y	N	Y	N	CD	NA	M
Basetti et al. (2020)	Y	Y	Y	Y	N	NA	Y	N	Y	Y	CD	Y	M
Osornio et al. (1996)	Y	Y	Y	Y	N	Y	Y	N	N	N	CD	Y	M

**Table 3:** Cure in cUTI, acute pyelonephritis, *E. coli* eradication, resistance, and adverse event.

Study ID	Wagenlehner et al., 2015	Kaye et al., 2018	Kaye et al., 2019	Seo et al., 2017	Arakawa et al., 2018	Risk ratio	95% CI
Intervention drug	Ceftolozane/tazobactam	Piperacillin/tazobactam	Piperacillin/tazobactam	Piperacillin/tazobactam	Ceftolozane/tazobactam		
Clinical cure in cUTI % (*n*/*N*)	67.1 (47/70)	92.1 (35/38)	41.57 (35/84)	N/A	72.9 (35/48)	1.21	1.00-1.47
Clinical cure in acute % (*n*/*N*) pyelonephritis % (*n*/*N*)	79 (259/328)	94.1 (95/101)	66 (62/94)	N/A	63.6 (14/22)	0.97	0.89-1.06
Microbiological eradication o *E. coli* % (*n*/*N*)	90.5 (237/262)	84.6 (154/182)	63.2 (84/133)	93.9 (31/33)	83.5 (66/79)	1.15	1.07-1.23
Resistance	2.7 (20/731)	18 (26/142)	3.3 (6/178)	N/A	N/A	0.25	0.14-0.45
Serious adverse events % (*n*/*N*)	2.8 (15/533)	4.8 (13/273)	2.6 (6/231)	N/A	11.4 (13/114)	1.15	0.64-2.09
Adverse effects % (*n*/*N*)	5.8 (31/533)	4.4 (12/273)	2.2 (5/231)	N/A	58.8 (67/114)	5.11	3.01-8.68

## Data Availability

All the data related to this study is presented in this study and attached supplementary materials.

## Supplementary Materials

Supplementary Materials Supplementary Materials Funnel plot graph 1A: standard error calculation for publication bias (Figure 1(a)). Forest plot of clinical cure in patients with cUTI after removal of publication bias. Black squares indicate proportion, and horizontal lines indicate 95% CI. Funnel plot graph 2A: after removing the publication bias (Figure 2(a)). Forest plot after removal of publication bias.
