# Supplementary material for: A Meta-Analysis on Clinical Outcomes of Ceftolozane versus Piperacillin in Combination with Tazobactam in Patients with Complicated Urinary Tract Infections
Source: Biomed Res Int. 2022 Aug 8;2022:1639114. doi: 10.1155/2022/1639114 (PMC9377909; doi:10.1155/2022/1639114)
Supplement: Supplementary Materials — Funnel plot graph 1A: standard error calculation for publication bias (Figure 1(a)). Forest plot of clinical cure in patients with cUTI after removal of publication bias. Black squares indicate proportion, and horizontal lines indicate 95% CI. Funnel plot graph 2A: after removing the publication bias (Figure 2(a)). Forest plot after removal of publication bias. [file 1639114.f1.docx]

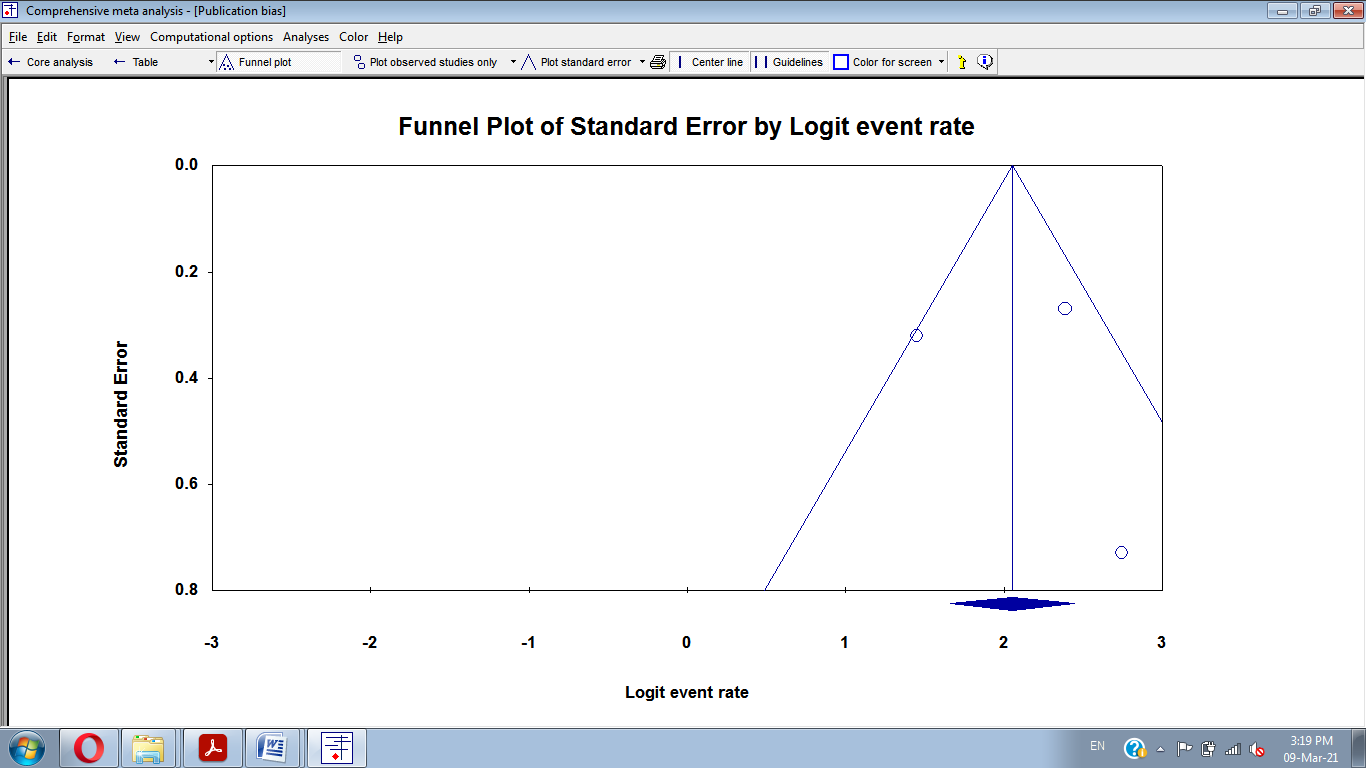


**Standard error**

| Study ID | Proportion of Clinical Success | Lower limit | Upper limit | Z value | p-value |  | Relative weight |
| --- | --- | --- | --- | --- | --- | --- | --- |
| *Kaye et al. 2019* | 0.916 | 0.865 | 0.949 | 5.275 | ≤0.001 | 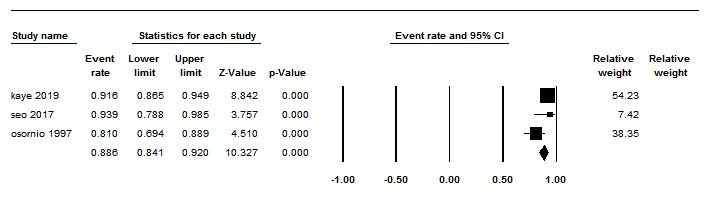 | 20.15 |
| *Seo et al. 2017* | 0.939 | 0.788 | 0.985 | 3.757 | ≤0.001 |  | 2.76 |
| *Osornio et al. 1997* | 0.810 | 0.725 | 0.955 | 3.785 | ≤0.001 |  | 14.25 |
| *TOTAL* | 0.886 | 0.841 | 0.920 | 10.327 | ≤0.001 |  |  |
| *Fixed effects model*  *heterogeneity τ=0.294 Diff Q=2, I2=66.542 P*≤*0.050* | | | |  |  |  |  |
|  |  |  |  |  |  |  |  |
| Fig 1A forest plot of clinical cure of in patients with cUTI after removal of publication bias Black squares indicates proportion, horizontal lines indicate 95%CI | | | | | | | |


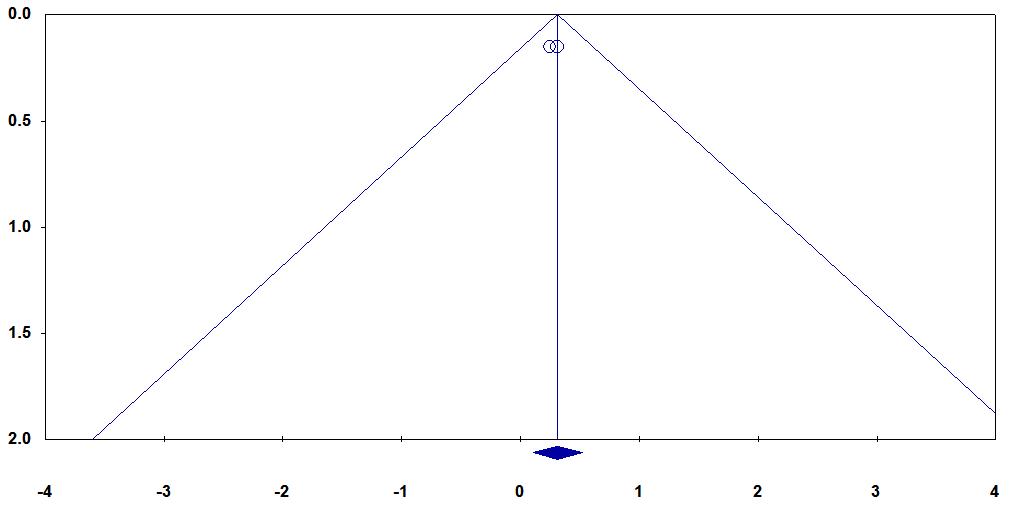


**Standard error**


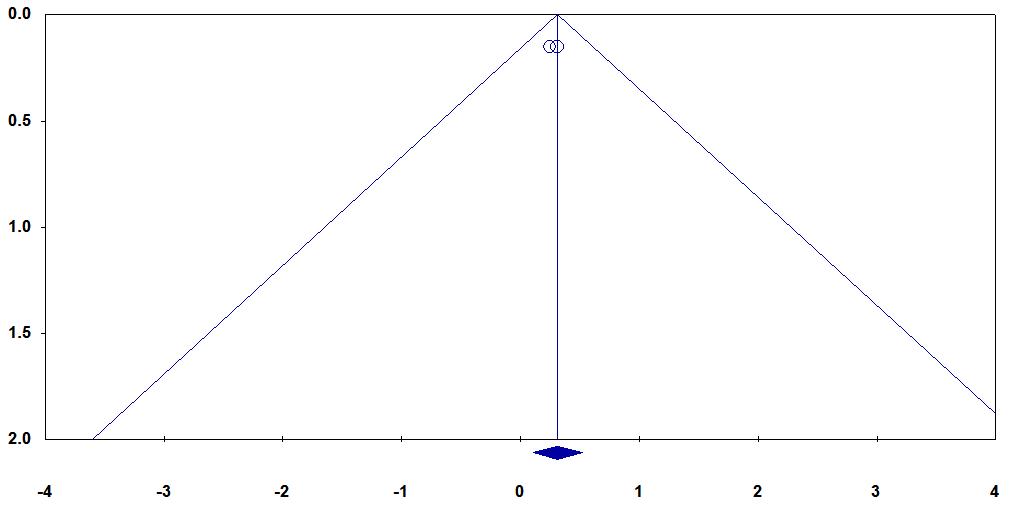


| Study ID | Proportion of Clinical Success | Lower limit | Upper limit | Z value | p-value | 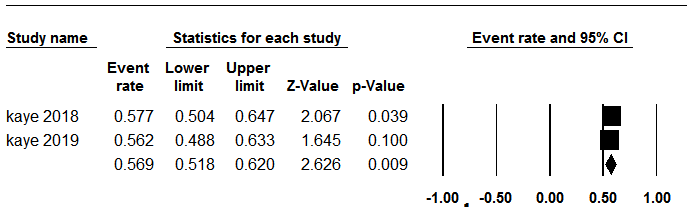 | Relative weight |
| --- | --- | --- | --- | --- | --- | --- | --- |
| *Kaye et al. 2018* | 0.577 | 0.504 | 0.647 | 2.067 | ≤0.039 |  | 45.19 |
| *Kaye et al. 2019* | 0.562 | 0.488 | 0.633 | 1.645 | ≤0.100 |  | 44.58 |
| *TOTAL* | 0.569 | 0.518 | 0.620 | 2.626 | ≤0.009 |  |  |
| *Fixed effects model*  *Heterogeneity τ=0.000 Diff Q=1, I2=0.000 P*≤*0.772* | | | |  |  |  |  |
| Fig A2. funnel plot after removal of publication bias | | | | | | | |


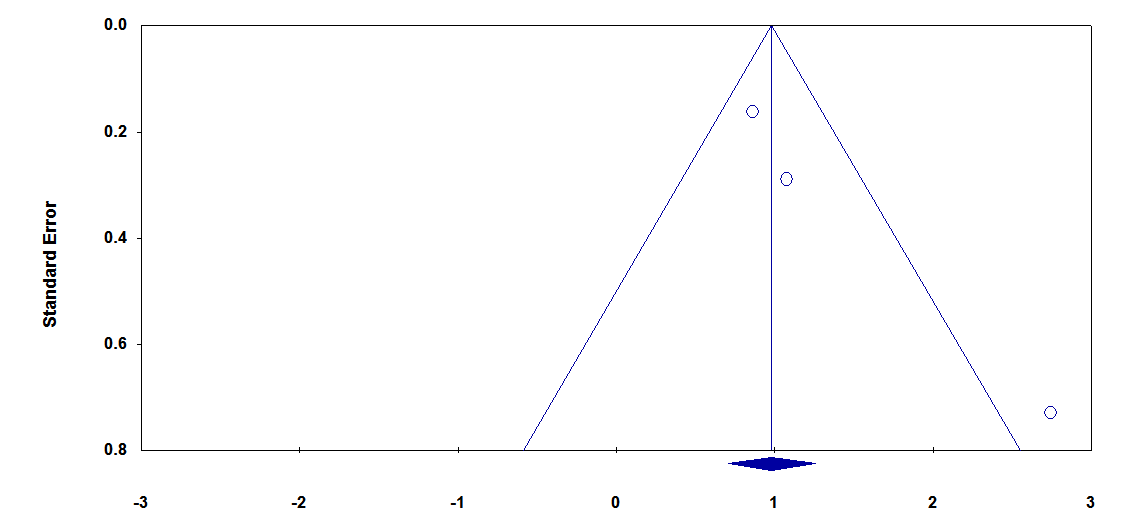


| Study ID | Overall clinical cure | Lower limit | Upper limit | Z value | p-value | 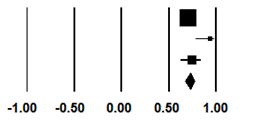 | Relative weight |
| --- | --- | --- | --- | --- | --- | --- | --- |
| *Kaye et al. 2018* | 0.703 | 0.633 | 0.765 | 5.319 | ≤0.001 |  | 39.59 |
| *Seo et al. 2017* | 0.939 | 0.788 | 0.985 | 3.757 | ≤0.001 |  | 1.96 |
| *Osornio et al. 1997* | 0.746 | 0.625 | 0.838 | 3.723 | ≤0.001 |  | 12.44 |
| *TOTAL* | 0.727 | 0.670 | 0.778 | 7.057 | ≤0.001 |  |  |
| *Fixed effects model*  *Heterogeneity τ=0.211 Diff Q=2, I2=69.032 P*≤*0.040* | | | | | |  |  |
|  |  |  |  |  |  |  |  |
| Fig A3. Forest plot for overall clinical success after 28 days of piperacillin/tazobactam in patients with cUTI after removal of publication bias. Black square indicates proportion, horizontal lines indicate 95%CI | | | | | | | |
